# Metastatic prostate cancer cell-specific phage-like particles as a targeted gene-delivery system

**DOI:** 10.1186/1477-3155-11-31

**Published:** 2013-09-23

**Authors:** Olusegun A Fagbohun, Robert A Kazmierczak, Valery A Petrenko, Abraham Eisenstark

**Affiliations:** 1 Cancer Research Center, Columbia, MO 65201, USA; 2 University of Missouri, Columbia, MO 65211, USA; 3 Department of Pathobiology, College of Veterinary Medicine, Auburn University, Montgomery, AL 36849, USA; 4 Department of Veterinary Microbiology and Parasitology, University of Ibadan, Ibadan, Nigeria

**Keywords:** Phage-like particles, Prostate cancer, Phage, Gene-delivery

## Abstract

**Background:**

One of the cardinal requirements for effective therapeutic management of tumors is the selective delivery of cancer drugs to the right site by ligand-decorated nanomedicines. Screening of 2 × 10^9^ clone landscape phage library provides a reliable avenue for generating protein ligands specific for tumor cells. It was shown that selective phage proteins derived from landscape phage libraries against breast and prostate cancer cells are able to navigate drug or siRNA loaded liposomes to corresponding cancer cells with minimal toxicity to non-neoplastic cells. In an alternative platform, glioma cell-specific phage proteins were used for assembling *in vivo* cancer-specific phage-like particles, named ‘phagemid infective particles’ as targeted gene-delivery vehicles.

**Methods:**

To extend the panel of anticancer cell phages, we have screened a 2 × 10^9^ clone landscape phage library f8/8 to select phage clones specific for metastatic prostate cancer cell PC-3M. The phage clones were characterized for their selective interaction with PC-3M cells using phage capture assay, immunofluorescence microscopy and electron microscopy. A prostate cancer selective phage was converted to phage-like particles harboring emerald green fluorescent protein.

**Results:**

Phage clone EPTHSWAT (designated by the sequence of inserted peptide) was found to be most selective for PC-3M cells and was observed to internalize PC-3M cells as revealed by immunofluorescence microscopy and electron microscopy. Conversion of this phage to phage-like particles harboring emerald green fluorescent protein and the expression of emerald green fluorescent protein in the phage-like particles treated PC-3M cells showed potential of adoption of this phage-like particle in prostate cancer therapeutic gene delivery.

**Conclusion:**

Successful employment of phage-like particles expressing emerald green fluorescent protein genes targeted to prostate cancer cells PC-3M confirms a prospect of their use for targeted delivery of therapeutic genes to cancer cells.

## Background

Prostate cancer is the second most frequently diagnosed cancer amongst men in the US. An estimated 238,590 new cases are estimated to be diagnosed with an expected death of 29,720 in men in the U.S in 2013 [1]. Conventional treatment modalities based on surgery, radiotherapy and chemotherapy have not ensured complete remission of the disease, which would account for 28% incidence of cancers of men in the U.S in 2013 [1]. Therefore, new concepts of prostate cancer care, including therapeutic oligo- and polynucleotides have been proposed as a promising alternative [2]. However, a prerequisite for effective gene therapy is specific delivery of therapeutic genes to a target tissue and its sustained expression in the effected cancer cells [3]. Current gene delivery approaches are based on the use of viral and non-viral delivery systems. Non-viral systems comprise naked DNA, or DNA associated with polymers, dendrimers, liposomes, polyethylenimine, polylysine, cationic peptides and, other non-infective vehicles. While non-viral delivery systems assure safety and modularity [4,5], they rank in efficiency of cell transduction below mammalian viral therapeutic oligonucleotides delivery vesicles based on herpesviruses, retroviruses, adenoviruses, or adeno-associated viruses, which have been evolved to acquire natural tropism for mammalian cells. The natural high transduction activity of viral vehicles, however can limit their use as therapeutics, since they can readily infect both target tumor cells and non-related healthy cells and can provoke fulminant immune response. Thus, they require reengineering to extirpate their natural tropism, re-target to specific tissues and make them less immunogenic [6,7]. By contrast, filamentous bacteriophages (phages) which are prokaryotic viruses, possess no natural tropism for mammalian cells, yet can be adapted to evolve tropism for target tumor cells by fusion of their surface proteins with tumor-specific peptides [8,9]. For example, phages with fusion peptides as targeting ligands on the pIII proteins have been shown to transduce mammalian cells with subsequent transgene delivery [10-13]. In another study, [14] developed phagemid particles with epidermal growth factors fused to pIII to transduce mammalian cells. Another type of display, with fusion peptides displayed on all multiple copies of the major coat protein pVIII can be even more advantageous for cell transduction since the multivalent display results in more strong binding of the phage to cellular receptors due to avidity effect, which provokes receptor dimerization and clustering [3,15,16]. Another type of tumor selective phagemid gene-delivery vehicles known as phagemid infective particle was designed by [17] using a phagemid system. These particles, ~ 700 nm long by ~10 nm in diameter, display tumor specific pVIII or pIII–fusion peptides which encapsulate a foreign phagemid DNA harboring all genetic elements necessary for expression of transgenes in mammalian cells [9]. Thus, the phage-targeted phagemid gene delivery can be considered as a new to tumor management strategy. In the light of this, we have screened a 1 × 10^9^clone landscape phage library to isolate metastatic prostate cancer-specific phage clones. The penetration of the most specific phage clone into mammalian cells was studied using fluorescence and electron microscopy. Thereafter, the phage clone was converted into phage-like particles using the phagemid system and its PC-3M cell-transforming activity was observed using emerald green fluorescent protein marker.

## Results and discussion

### Selection of prostate cancer cell-interacting phage clones and studying their selectivity towards the target cells

To generate peptides specific for prostate cancer cells, landscape phage library with an octapeptide insert was screened against the metastatic prostate cancer cell line PC-3M involving two selection rounds. 18 clones were randomly picked and the structures of the displayed peptides were determined using PCR, sequencing and translation of the DNA sequences into amino acids using DNASTAR software (DNASTAR, Madison, WI) (Table 1). Phage clone EPTHSWAT was the most frequently selected, occurring 8 times, while phage clone GDIVTSNS occurred 7 times.

**Table 1:** Phage clones generated from the second round screening of the f8/8 landscape phage library

**Fusion peptides**	**Frequency**
EPTHSWAT	8
GDIVTSNS	7
DSNGTSTQ	1
EDEEPSTS	1
DVTTGAYS	1

Phage clones are designated by the structure of the inserted (fused) guest peptides.

**Figure 1:**
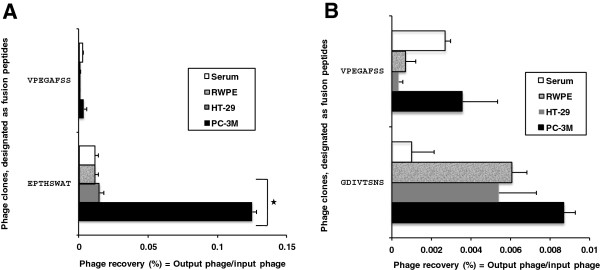
**Selectivity of the phage clones EPTHSWAT and GDIVTSNS toward target cells PC-3M in comparison with other cells (non-neoplastic prostate epithelial cells, RWPE-1; colon adenocarcinoma cells, HT-2A) and serum.** Panel **A** depicts levels of interaction of phage EPTHSWAT with PC-3M cells, RWPE cells, HT-29 cells and serum; Panel **B** shows the levels of phage GDIVTSNS interaction with PC-3M cells, RWPE cells, HT-29 cells and serum. Selectivity was estimated as phage recovery (%) = output (cell-associated) phage/input phage. Unrelated phage bearing the guest peptide VPEGAFSS was used as a control.

Selectivity of the two abundant prostate cancer-specific phages EPTHSWAT and GDIVTSNS towards PC-3M cells were studied in a phage capture assay (Figure 1). Selectivity assay is based on interaction of phage particles with PC-3M cells in comparisons with other cells RWPE-1 (non-neoplastic prostate cells), HT-29 (human colon carcinoma cells) and serum. As seen in Figure 1, phage EPTHSWAT showed more selective interaction with PC-3M cells with 14.4 fold greater interaction than the phage GDIVTSNS and 35.1 fold greater than the non-relevant control phage VPEGAFSS. Furthermore, phage EPTHSWAT showed a statistically significant higher interaction with PC-3M cells than with other cells RWPE-1, HT-29 and serum. This high interaction of the phage particles with PC-3M cells might be due to phage interaction with overexpressed macromolecules on the surfaces of the cells.

### Selective interaction of phage EPTHSWAT in a co-culture with PC-3M, RWPE-1 and HT-29 cells

To further confirm the selective interaction of phage EPTHSWAT with PC-3M cells;PC-3M, RWPE-1 and HT-29 cells were co-cultured and incubated with phage EPTHSWAT for 1 h at 37°C. Phage was fluorescent labeled with antibodies and observed by fluorescence microscopy.

**Figure 2:**
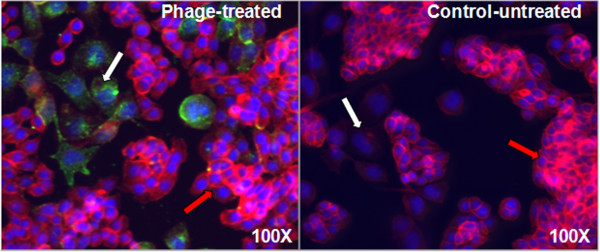
**Immunofluorescence microscopic demonstration of phage clone EPTHSWAT selectivity towards PC-3M cells in a co-culture of PC-3M, RWPE-1 and HT-20 cells.** White arrows point PC-3M (prostate cancer) cells whereas red arrows point to HT-29 (colon cancer) cells. Phage EPTHSWAT intra-cytoplasmic and nuclear localization at 1 h post-incubation with the co-culture of PC-3M and HT-20 cells. Phage particles stained with goat anti-rabbit Alexa Fluor 488 were revealed as abundant green spots in PC-3M cells in comparison with the very scanty phage particles in HT-29 cells. The control uninfected co-culture showed no green spots.

We observed that majority of the phage particles localized in PC-3M cells with very scanty amount in RWPE-1 and HT-29 cells. As shown in Figure 2, the predominant interaction of the phage particles with PC-3M cells is auspicious for harnessing the phage peptides to navigate therapeutics to the desired site of lesion with minimal bystanders’ effect.

**Figure 3:**
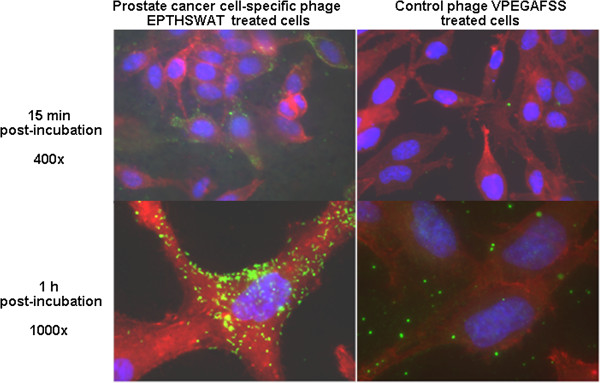
**Immunofluorescence microscopic demonstration of phage clone EPTHSWAT interaction with PC-3M cells at two time points 15 min or 1 h in comparison with the control non-relevant phage VPEGAFSS.** Phage particles were revealed as green spots and linear particles in the cytoplasm and nuclei of the cells at 40X and 100X magnification of the objective lens at 15 min and 1 h post-incubation, respectively.

The selective interaction of phage EPTHSWAT with PC-3M cells in comparison with the non-relevant phage VPEGAFSS at 15 min or 1 h was also studied (Figure 3). At 15 min post incubation, phage EPTHSWAT was already interacting with PC-3M cells while the control phage particles were washed away during the washing stage of the procedure. At 1 h post-incubation, a great amount of phage EPTHSWAT were already bound or internalized by PC-3M cells whereas very scanty control phage particles were observed interacting with the cells. The interaction of phage EPTHSWAT with the cells at the two time points were specific because phage particles still interacted with the cells despite stringent washing steps employed in the procedure. This is also evidenced by the washing away of majority of the control phage particles bound to the cells through non-specific interactions.

### Tracking phage EPTHSWAT interaction with PC-3M cells

**Figure 4:**
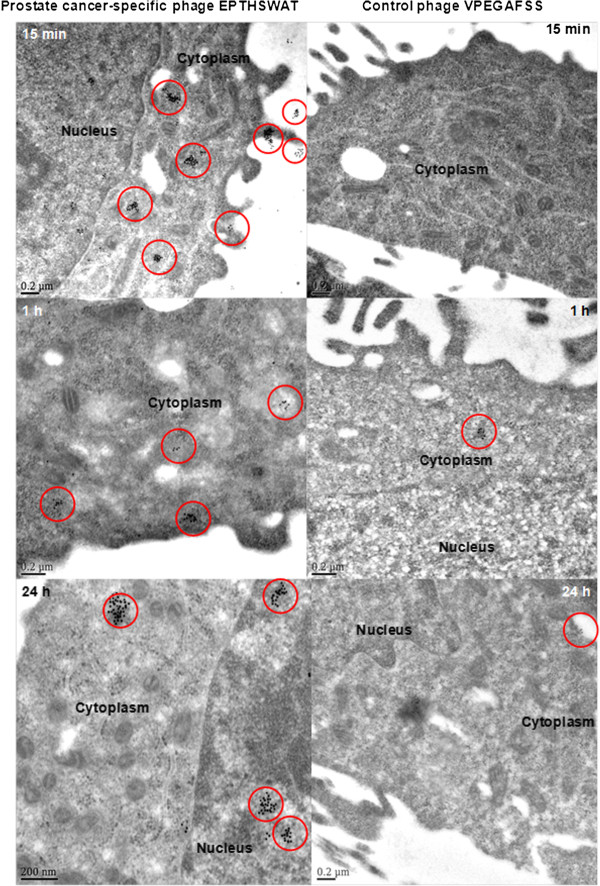
**Electron micrographs of thin sections of phage EPTHSWAT–PC-3M cells and the control phage VPEGAFSS–PC-3M cells interactions.** The localization of phage particles is revealed by the presence of gold particles in the red circles. At 15 min post-incubation, a lot of phage EPTHSWAT particles had bound and internalized PC-3M cells whereas phage VPEGAFSS had been washed off. At 1 h post-incubation, more phage EPTHSWAT particles were seen internalizing PC-3M cells than the control phage. At 24 h post-incubation, more EPTHSWAT particles had internalized and homed into the nucleus in comparison with the control phage.

Immunogold electron microscopy was employed to precisely track the kinetics of phage EPTHSWAT interactions with PC-3M cells in comparison with the control phage VPEGAFSS.Phage EPTHSWAT was incubated with PC-3M cells at three time points 15 min, 1 h or 24 h. As seen in Figure 4, at 15 min post-post incubation, phage EPTHSWAT particles had already bound to the surface of the cells and some had gain entry into the cytoplasm, whereas, the control phage particles were not observed interacting with the cells.

The binding and internalization of phage EPTHSWAT particles at this time point corroborates the previously described results of immunofluorescence tracking of phage interaction with PC-3M cells. This further revealed the selective interaction of the test phage interacting with PC-3M cells in comparison with the control phage. Absence of the control phage particles interacting with PC-3M was as a result of the washing step in the procedure whereby non-specific interacting phage particles were washed off. The internalization of phage EPTHSWAT into the cytoplasm at 15 min post-incubation might be applicable for harnessing the phage fusion peptides for rapid targeted delivery of therapeutics. Based on the binding and internalizing of the test phage at 15 min post-incubation, it could be inferred that the phage mechanism of entry into PC-3M cells is not through endocytic pathway. This is also supported by the fact that the length of the phage particle (1 μm) will not fit into the membrane pits 700 nm in depth [18].

### Generation of phage-like particles

**Figure 5:**
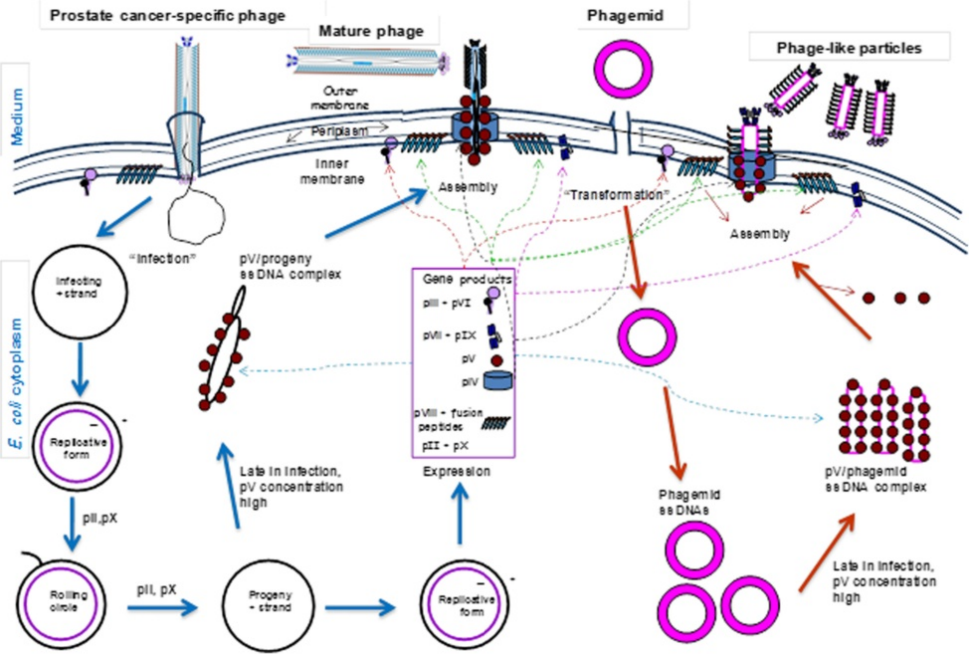
**Schematic representation of the rescue of prostate cancer specific phage-like particles by the prostate cancer-specific helper phage EPTHSWAT in E. coli.** The blue arrows indicate the replication cycle of the helper phage generating proteins needed for replication and encapsidation of the phagemid, whereas, the maroon arrows show the replication and rescue of phage-like particles. The dotted lines indicate the translocation of the helper phage expressed proteins to the inner membrane where they are involved in assembly. pV binds ss DNA whereas pII nicks ds DNA and pX acts as inhibitor of pII function.

To generate phage-like particles, K91 blue *E. coli* cells were transformed with pcDNA™6.2-GW/EmGFP-miR vector encoding emerald green fluorescent protein (EmGFP). The phagemid encodes EmGFP under the control of a Pol II human CMV (cytomegalovirus) promoter and Herpes simplex virus (HSV) thymidine kinase (TK) polyadenylation signal; f1, pUC and SV40 origins of replication; spectinomycin (*aad*A1) and blasticidin (*bsd*) resistance genes. The phagemids were rescued with phage EPTHSWAT (helper phage) as illustrated (Figure 5).

### Transmission electron microscopic imaging and quantification of phage-like particles and phage

**Figure 6:**
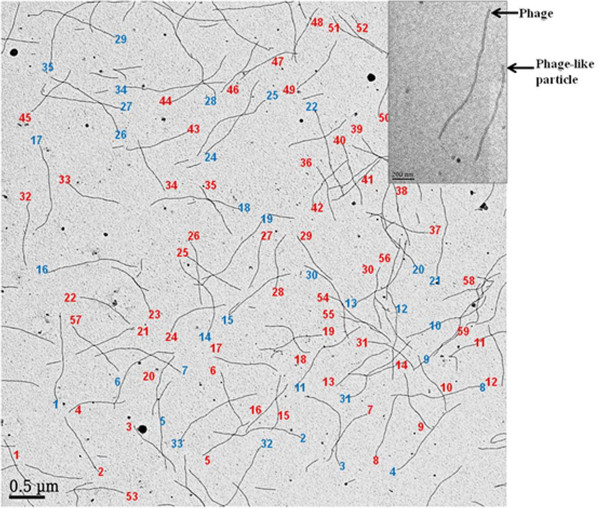
**Negative contrast electron micrograph of phage-like particles and phage EPTHSWAT.** The phage-like particles are numbered in red whereas the phage particles are in blue. The number of phage-like particles on the shown field is 59 whereas the phage particles are 36, therefore the ratio of phage-like particles to the phages is 1.6. The phage-like particle is about 2/3rd the size of the phage (Inset).

Transmission electron microscopy (TEM) was employed to visualize the mixture of phage-like particles and phage EPTHSWAT, quantify the particles and estimate their lengths. As anticipated, some phage EPTHSWAT were produced along with the phage-like particles but the number of phage-like particles superseded that of the phage particles due to the vector low copy number and defective assembly of the phage particles created by disruption of the origin of the minus-strand synthesis with the insertion of the Tn10 fragment [19]. The number of phage-like particles on the electron microscopic field was 59 whereas the phage particles were36; therefore the molecular ratio of phage-like particles to the phages is 1.6 as shown (Figure 6).

Virions/ml = (A_269_ (6 × 10^16^)/ nucleotides in the genome, where A_269_ represents an absorbance at 269 nm [20]. Therefore, for phage-like particles with the genome size 5759 nucleotides, 1 AU_269_=1.04 × 10^13^ virions/ml whereas for the phage EPTHSWAT with genome size 9198 nucleotides, 1 AU_269_=6.5 × 10^12^ virions/ml. The molecular ratio of phage-like particles to phage particles is 1.6, as determined by TEM (Figure 6). Thus their mass ratio is (5759 × 1.6)/9198 = 1. Thus, 1 AU_269_ of preparation contains 0.5 × 1.04 × 10^13^= 5.2 × 10^12^ virions/ml of phage-like particles and 0.5 × 6.5 × 10^12^ = 3.25 × 10^12^ virions/ml of phage particles. The phage-like particle is about 2/3^rd^ the size of the phage, since the calculated length of the phage-like particle is 843 nm [(1.435 Ǻ X 5759) + 175 Ǻ] and that of phage EPTHSWAT is 1337 nm [(1.435 Ǻ X 9198) + 175 Ǻ] [21] as confirmed (Figure 6).

### *in vitro* expression of emerald green fluorescent protein gene

**Figure 7:**
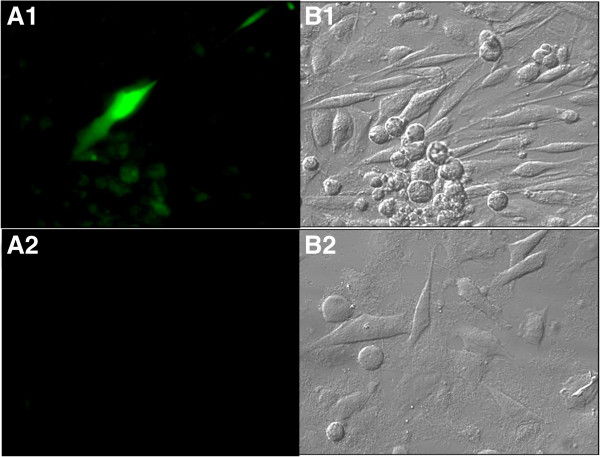
***in vitro *transduction of phage-like particles and expression of EmGFP. A1** and **B1** represent PC-3M cells incubated with phage-like particles and phage at 37°C for 48 h and then observed with FITC filter epifluorescence and bright field, respectively. **A2** and **B2** show cells incubated with phage particles only and observed with FITC filter epifluorescence and bright field, respectively.

To track the expression of expression of emerald green fluorescent protein gene in the phage-like particles; phage-like particles in combination with the phage were incubated with PC-3M cells at 37°C for 48 h while cells incubated with phage only served as the control. Cells were washed with PBS, fixed and visualized by epifluorescence microscopy. As seen in Figure 7, some cells incubated with phage-like particles and phage demonstrated emerald green fluorescent protein expression as revealed by the green fluorescence when observed with FITC filter of the epifluorescent microscope (1A) whereas no fluorescence was observed with the control (2A).

## Conclusion

In this study, we demonstrated that screening of a 2 × 10^9^ landscape phage library against metastatic prostate cancer cell PC-3M allows isolating highly selective prostate cancer cell-specific phage, which can be readily converted into a phage-like particle transducing the mammalian prostate tumor with the potential to deliver therapeutic genes and their expression products to cancer cells leaving normal cells unscathed. Notwithstanding, the procedure was carried *in vitro* and peptide selectivity to towards prostate tumor has not been tested *in vivo*; the peptides generated from the screening procedure might home tumor cells when tested *in vivo* based the stringent steps involved in the selection procedure. Furthermore, peptides generated from screening landscape phage library proffers avidity effect based on multivalency that can arouse receptor dimerization and clustering [3,15,16] in comparison with commonly screened p-III fusion libraries with affinity features. The delivery and expression of emerald green fluorescent protein was revealed by visualization of green fluorescence observed in some of the treated PC-3M cells. Although the expression of the reporter gene EmGFP was not high; this could have been improved if adeno-associated virus inverted terminal repeats were present in the phagemid vector as demonstrated [8]. The results of this study can pave a way for developing highly efficient transgene or oligonucleotides delivery systems against other maladies aside cancer. Screening of landscape phage libraries provides an effective way for development of highly selective ligands to a variety of cell receptors and their easy conversion into non-pathogenic phage-like particles, which are promising vectors for delivery of various gene pharmaceuticals.

## Methods

### Cell lines

All cell lines were purchased from the American Type Culture Collection (ATCC, Manassas, VA, USA)_._Cell line PC-3M was employed as the target cell, whereas WI-38 (CCL-75) normal human lung fibroblasts were used for depletion of phage library. Cells were cultivated in 25 cm^2^ cell culture flasks (Corning Inc., Corning, NY) containing their respective media with L-glutamine (Sigma-Aldrich, St. Louis, MO) supplemented with 10% fetal calf serum (FCS) at 37°C in 5% CO_2_ cultivated.

### Phage display library

The landscape phage library f8/8 containing 2 × 10^9^ clones [22] was screened to isolate clones binding target prostate cancer cell lines. All procedures for handling phages, including propagation, purification, titering, isolation of phage clones, and isolation of phage DNA have been described [23]^.^*Escherichia coli* (*E. coli*) strain K91Blue {Hfr C thilacZΔ M15 lac Y::mkh lacI^Q^} used for propagating phages was kindly provided by George Smith [24].

### Selection of prostate cancer cell-interacting phage clones

Screening of the phage library against PC-3M target cells was run in T-25 cell culture flask. Briefly, an aliquot of the phage library containing 100 billion phage particles in serum-free medium was incubated in an empty cell culture flask at 37°C for 1 h to deplete phage particles binding plastic. Unbound phages recovered from the depletion were incubated with pre-incubated serum in cell culture flask at 37°C for 1 h to deplete phage particles binding serum. Thereafter, unbound phage particles were incubated with confluent fibroblasts at 37°C for 1 h to deplete phage particles interacting with fibroblasts. Unbound phages were removed, added to the target cells and incubated at 37°C for 1 h. Subsequently, unbound phages were discarded and cells were washed ten times with washing buffer (0.1% BSA, 0.1% Tween 20 in serum free medium) to remove any remaining unbound phage particles. Targeted cell-bound phage particles were eluted with elution buffer (0.1 M glycine-HCl, pH 2.2) for 10 min on ice and neutralized with 1 M Tris–HCl (pH 9.1). Phage particles in eluate were concentrated in Amicon ultra-4 concentrators as recommended by the manufacturer (Millipore Corp, Bedford, MA)and used in subsequent rounds of selection but with no depletion steps. The amplified eluate was as input in further rounds of selection. These procedures were similar to the procedure described above but with the exclusion of the depletion steps depletion steps. Two rounds of selection were performed. In each round, the enrichment of phage particles binding to the cells was determined by titering of input and output phages. The ratio of output to input phage increased from one round to another indicating successful selection of phage clones interacting with the target cell lines.

### Testing of phage clones for selectivity towards prostate cancer cells

PC-3M cell-specific phage clone EPTHSWAT was characterized for their selectivity toward target prostate cancer cell PC-3M in comparison with control cells RWPE (non-neoplastic prostate epithelia), HT-2A (colon carcinoma) and serum in a phage capture assay. Briefly, target cells PC-3M, and control RWPE and HT-2A cells were cultivated in triplicate to confluence in separate wells of 96-well cell culture plates. The medium with serum was incubated in separate wells in triplicate as a control. Cells incubated with serum-free medium at room temperature for 1 h were incubated with phage (1 × 10^6^ cfu) at room temperature for 1 h. Unbound phages were carefully removed and cells were washed with 100 μl washing buffer for 5 min eight times to remove non-interacting phages. Cells were lysed with 25 μl lysis buffer (2.5% CHAPS) for 10 min on a shaker. The lysate containing cell-interacting phages were titered in *E. coli.* Phage recovery was calculated as the ratio of output phage to input phage. An unrelated phage with non-relevant guest peptide VPEGAFSS was used as the control (Figure 1).

### Immunofluorescence analysis of the selectivity of phage EPTHSWAT towards PC-3M cells

PC-3M, HT-29 and RWPE cells were co-cultured in Lab-Tek™ 4-well chamber slides (Thermo Scientific, Rochester, NY) until they were 60% confluent. Subsequently, the cells were incubated with 1 × 10^11^ phage particles in RPMI 1640 medium for 1 h at 37°C while the other cells served as the uninfected controls. Thereafter, cells were washed with PBS (pH 7.4), fixed in 4% paraformaldehyde for 10 min, permeabilized with 0.25% Triton X-100 (Sigma, St. Louis, MO) in PBS for 10 min and blocked with 5% goat serum (MP Biomedicals, Solon, OH) for 1 h. Cells were treated with 1:1000 dilution of 5 mg/ml rabbit anti-fd bacteriophage antibodies (Sigma-Aldrich, St. Louis, MO) for 1 h, washed and incubated with 1:500 AlexaFlour® 488 anti-rabbit IgG, 0.022 μM Alexa Flour® 546 phalloidin (Molecular Probes, Carlsbad, CA) for 1 h. Subsequently, cells were washed three times with PBS and covered with VECTASHIELD mounting medium with DAPI (Vector Laboratories, Burlingame, CA). Images were acquired in grayscale with a 12-bit digital camera coupled to Olympus BX51 microscope (Olympus, USA) equipped with bandpass emission fluorescence filter optical blocks. During the processing stage, individual image channels were pseudocolored with RGB values corresponding to each of the fluorophore emission spectral profiles with the exception of Alexa Flour® 546 phalloidin, which was pseudocolored red (Figure 2). Interaction of phage EPTHSWAT with PC-3M cells in comparison with the non-relevant phage VPEGAFSS was used to further confirm the selectivity of phage EPTHSWAT towards PC-3M cells. 1 × 10^11^ phage EPTHSWAT and phage VPEGAFSS were incubated with their corresponding confluent PC-3M cells at 37°C for 15 min or 1 h and processed as described previously and images were acquired with the Olympus BX51 microscope (Figure 3).

### Immunogold electron microscopic analysis of the selectivity of phage EPTHSWAT towards PC-3M cells

PC-3M cells were seeded into Thermonax cover slips (Thermo Scientific, Rochester, NY) and grown until confluence. 1 × 10^11^ of phage EPTHSWAT particles were incubated with cells at three time points: 15 min, 1 h or 24 h at 37°C. For the 24 h incubation, cell culture growth medium was replaced with new growth medium at 1 h post-incubation to remove unbound phage particles and cells were thereafter incubated for 24 h. The non-relevant phage VPEGAFSS served as the negative control. At the end of incubation for the three time points, cells were washed thrice with PBS to remove unbound phage particles, fixed in 2% paraformyladehyde, 1% glutaraldehyde in 0.1 M cacodylate, pH 7.4., post-fixed in 1% tannic acid (Electron Microscopic Sciences, Hatfield, PA), dehydrated in ethanol and infiltrated and embedded in LR White resin (Electron Microscopic Sciences, Hatfield, PA). Ultrathin sections were cut on formvar-carbon coated nickelgrids and labeled as described [25] with 1:50 rabbit anti-fd bacteriophage IgG (Sigma-Alrich, St. Louis, MO) and 1:5 goat anti-rabbit conjugated with 10-nm gold particles. Grids were rinsed and post-stained with 0.5% aqueous uranyl acetate. Samples were observed in Joel 1400 electron microscope operated at 80 kV.

### Generation of phage-like particles

pcDNA™6.2-GW/EmGFP-miR vector was used to transform electrocompetent K91 blue *E. coli* and transformants were selected on NZY plates containing 50 μg/ml spectinomycin grown at 37°C for 16 h. Transformants were picked and propagated in 2 ml NZY media containing 50 μg/ml spectinomycin with shaking at 200 rpm at 37°C overnight. Subsequently, 300 μl overnight cultures was added to 20 ml of NZY plates containing 50 μg/ml spectinomycin and grown at 37°C in a shaking incubator at 200 rpm until the OD reached 0.450 at 600 nm. Thereafter, the culture was inoculated with 1.0 × 10^9^ colony forming units (cfu) of the prostate cancer-selective phage EPTHSWAT and incubated at 37°C for 15 min at 50 rpm for the phage to infect the *E. coli.* Then, 0.2 μg/ml of tetracycline was added to the culture and incubated at 37°C for 40 min at 200 rpm. Following, tetracycline was added to 20 μg/ml and the culture was grown overnight. Finally, the phage-like particles preparation were collected by two successive polyethylene glycol 8000 precipitations and suspended in 100 μl Tris buffered saline (TBS; 50 mM Tris–HCl, pH 7.5 and 150 mM NaCl).

### Transmission electron microscopic imaging and quantification of phage and phage-like particles

6 μl of a mixture of phage-like particles and phage were dispensed on a carbon film coated copper grid (Electron Microscopic Sciences, Hatfield, PA) and incubated for 5 min at room temperature. The mixture was tipped off and the grid was stained with a drop of 5% uranyl acetate for 5 min at room temperature. The stain was tipped off and the grid was allowed to dry for 5 min and observed in Joel 1400 electron microscope operated at 80 kV. The quantities and ratio of phage-like particles and phage in the preparation were determined by counting their numbers in a field of view as depicted (Figures 6 and 7).

### *in vitro* expression of emerald green fluorescent protein

To test the expression of emerald green fluorescent protein in PC-3M cells, 8.0 × 10^10^ phage-like particles with 5.0 × 10^10^ phage EPTHSWAT were incubated with confluent PC-3M cells in the well of a Lab-Tek™ 4-well chamber slide (Thermo Scientific, Rochester, NY) for 48 h at 37°C while cells incubated with only 5.0 × 10^10^ phage EPTHSWAT served as the control. Cells were washed three times with PBS, fixed, visualized and imaged under Olympus BX51 microscope (Olympus, USA) with FITC emission fluorescence filter and bright field (Figure 7).

## Competing interests

The authors declare that they have no competing interests.

## Authors’ contributions

AE was the principal investigator and takes primary responsibility for the paper. VAP and OAF were instrumental in the design of the study. OAF performed the laboratory work for this study. AE, VAP and RAK participated in reviewing and editing the manuscript. All authors read and approved the final manuscript.
